# STEAM education and environmental ethics awareness among students aged 10-14 from an international perspective: a comparative analysis of cultural and educational philosophy factors

**DOI:** 10.12688/openreseurope.21790.1

**Published:** 2026-04-10

**Authors:** Elena Arce, Oya Güler

**Affiliations:** 1 Department of Industrial Engineering. Ferrol Polytechnic School of Engineering, University of A Coruna, Ferrol, La Coruña, 15403, Spain; 2 Faculty of Humanities and Social Sciences, Bursa Technical University, Bursa, Bursa, 16190, Turkey

**Keywords:** STEAM education; environmental ethics; sustainability education; cross-national comparison; compulsory education.

## Abstract

**Background:**

Environmental ethics awareness is a key component of sustainability education, and STEAM (Science, Technology, Engineering, Arts, and Mathematics) is often promoted as a lever to foster values-based environmental learning. Cross-national evidence for early adolescents remains limited.

**Methods:**

We surveyed 283 students aged 10–14 in compulsory education from Portugal, Turkey, and Poland participating in the Erasmus+ “Eco-Maker” project (KA220-SCH). Environmental ethics awareness was measured with a 23-item, four-factor Likert scale (α = .95). We conducted one-sample t tests (vs. the neutral point) and MANOVA to test gender and country differences, complemented by documentary analysis of national curricula and policy frameworks.

**Results:**

Students in all three countries reported high environmental ethics awareness across the four dimensions (all p < .001; very large effects). No significant differences were found by gender. Country differences were significant: Portuguese students scored higher on Definition of Environmental Ethics, Reasons for the Emergence of Environmental Ethics, and Purpose of Environmental Ethics, while no country differences were observed for Environmental Ethics Measures. Documentary analysis indicated that Portugal embeds sustainability, citizenship, creativity, and ethical responsibility across subjects; Poland emphasizes scientific and technological competence with project-based work but a more traditional STEM orientation; and Turkey frames STEAM around technological development and national climate targets, with uneven integration of arts and ethical discourse.

**Conclusions:**

Environmental ethics awareness among 10–14-year-olds is broadly high, but its emphasis and framing vary across educational and cultural contexts. Culturally grounded, STEAM-informed sustainability education and further research on measurement equivalence and behavioral outcomes are recommended.

## Plain Language summary

This study analyzes how young students think about the environment and their responsibility to protect it. We surveyed 283 students aged 10 to 14 in Portugal, Turkey, and Poland, all from schools that take part in an international Erasmus+ project on climate change and STEAM education (science, technology, engineering, arts, and mathematics).

Students answered questions about four main ideas: what environmental ethics means, what actions should be taken to protect the environment, why environmental ethics matters, and what the goal of environmental responsibility is. In general, students in all three countries strongly agreed that people should care for the planet and act in a sustainable way.

Girls and boys gave very similar answers. This suggests that environmental awareness is shared across gender at these ages. However, we did find differences between countries. Students in Portugal tended to give even stronger pro-environment answers than students in Turkey and Poland.

To help explain this, we also reviewed national education policies and school curricula. In Portugal, ideas like sustainability, citizenship, and ethical responsibility are clearly included across different subjects. In Poland, schools focus strongly on science and technology, often through practical projects. In Turkey, environmental issues are linked to national goals for cleaner technology and future climate targets. These results suggest that what students believe about the environment is closely related to what and how they are taught in school.

## 1. Introduction

In the complex and rapidly changing world of the 21st century, environmental problems have emerged as one of the most significant challenges confronting humanity. From climate change to deforestation, air pollution to biodiversity loss, the scale and urgency of these issues demand immediate and collective action. According to the World Health Organisation's 2021 report, problems such as air pollution, depletion of water resources, climate change and loss of biodiversity threaten not only the natural environment but also human health and quality of life ( World Health Organization, 2021). As stated in UNESCO's "Education for Sustainable Development" programme, increasing environmental awareness and acquiring the values necessary for a sustainable future should be one of the main goals of education systems ( UNESCO, 2024). In this context, Science, Technology, Engineering, Arts and Mathematics (STEAM) education stands out as an important tool in developing this awareness and competence ( UNESCO, 2023). However, the effectiveness of this process depends on a number of factors influenced by different cultural and educational philosophies.

STEAM education draws attention as a holistic education model that aims to develop students' 21st century skills such as critical and creative thinking, responsible innovation and computational thinking ( Quigley and Herro, 2016). However, STEAM education encourages not only interdisciplinary knowledge acquisition but also students' awareness of issues such as environmental sustainability, social justice and ethical values ( Komatsu et al., 2021). The Sustainable Development Goals (SDGs) adopted by the United Nations in 2015 emphasise inclusion, equity and lifelong learning opportunities in education ( United Nations, 2015). In particular, SDG4 states that quality education is the cornerstone of sustainable development and includes the development of environmental ethics and sustainability awareness ( Organisation for economic co-operation and development (OECD), 2025). In this direction, STEAM education offers a learning environment that allows students to develop innovative and creative solutions to environmental problems ( Perignat and Katz-Buonincontro, 2019)

According to Johnson and Zhang's research, especially students between the ages of 10-14 are in a critical period when their attitudes and behaviours towards the environment are shaped. In this context, STEAM education, integrated with environmental education, stands out as an effective tool to improve students' understanding of environmental ethics ( Lee, 2021; Lee and Anderson, 2022). For a sustainable world of the future, it is of great importance to develop young generations' understanding of environmental ethics. STEAM education stands out as an effective tool in this process and increases students' sensitivity to environmental problems. The role of different cultural and educational systems in this process should be taken into account in shaping educational policies. Thus, important steps will be taken to increase environmental awareness on a global scale and to build a sustainable future.

The main aim of this paper is to comparatively analyse the state of environmental ethics awareness among students aged 10-14 years in the context of three different countries (Portugal, Turkey and Poland). The study addresses aspects such as understanding of the definition of environmental ethics, students' attitudes towards environmental ethical measures, views on the reasons for its emergence and the perceived purpose of environmental ethics. In this context, the effects of cultural and educational context on environmental ethics are carefully considered and differences between countries are revealed.

The main purpose of this research is to examine the factors affecting students' environmental ethical awareness. In this context, the following research questions were determined:

1. Do students' environmental ethical awareness levels show a significant difference according to gender?
2. Do students' environmental ethical awareness levels show a significant difference according to the country variable?
3. What are the effects of educational systems and cultural approaches in different countries on students' perception of environmental ethics?
4. How does the level of emphasis of environmental ethics education in the curriculum differ across countries?
5. How do cultural, social and educational factors influence the development of students' environmental ethical awareness?

Within the framework of these research questions, the study will comprehensively examine the factors affecting the environmental ethics awareness of students in different countries. It will provide an important basis for understanding how environmental ethics education and awareness differ in the international context. Considering the global nature of environmental issues, such comparative research can contribute to raising public awareness and developing more effective educational policies. Furthermore, this analysis aims to provide suggestions on how STEAM education can be designed within the framework of ethical and value-based approaches ( Aguayo et al., 2023).

## 2. Literature review

### 2.1. STEAM education and environmental ethics

STEAM (Science, Technology, Engineering, Arts and Mathematics) education stands out as an interdisciplinary approach developed to respond to the global educational challenges of the 21st century. This education model is based on four basic principles such as creativity, inclusiveness, citizenship and emerging technologies ( Quigley and Herro, 2016). STEAM education enables students to develop 21st century skills such as critical and creative thinking, responsible innovation and computational thinking ( English and Gainsburg, 2016.). In this context, STEAM education is seen as a tool to support individual, societal and planetary well-being in line with the UNESCO 2030 Sustainable Development Goals ( UNESCO, 2025).

STEAM education is a multidisciplinary approach that aims to develop students' critical thinking, problem solving and creative expression skills ( Larson and Miller, 2011). The inclusion of art as well as science and mathematics increases students' interest in the subject and makes learning processes more interactive ( Eom et al., 2016). STEAM methods help students solve environmental problems by using hands-on learning tools. For example, with robotics and coding projects, students can develop innovative solutions on topics such as waste management systems or energy efficiency.

Environmental ethics is a discipline that deals with people's relations with the environment and their responsibilities for using natural resources ( Palmer, 2002). Environmental ethics awareness is an important factor that shapes individuals' attitudes and behaviours towards the environment. Studies show that environmental ethics education positively affects individuals' attitudes and behaviours towards environmental problems ( Surmeli and Saka, 2013). Especially students between the ages of 10-14 are in a critical period in the development of environmental ethical awareness. Environmental ethical values acquired in this age group enable individuals to develop lifelong sustainable attitudes ( Chawla, 1999).

STEAM education offers an effective platform for the development of environmental ethical awareness. By offering an interdisciplinary approach, STEAM education aims to improve students' problem-solving skills and increase their critical thinking abilities ( Lee, 2021). For example, STEAM projects allow students to generate solutions to real-world problems, while at the same time helping them to internalise the principles of environmental ethics. These projects emphasise ethical design principles, especially when working with disadvantaged, indigenous and underrepresented communities ( Aguayo et al., 2023).

Research reveals that teachers' pedagogical knowledge and skills need to be improved in order to implement environmental ethics education effectively ( Preston, 2011). Teachers' lack of sufficient knowledge about environmental ethics education may prevent the effective implementation of this education in the classroom environment ( Şahin and Halil-Kazoglu, 2017). Therefore, environmental ethics education should be included in teachers' pre-service and in-service training programmes.

As a result, STEAM education is strongly linked to environmental ethics and increases students' awareness of environmental issues, enabling them to acquire the necessary skills for a sustainable future. In this context, STEAM education not only provides technical knowledge and skills, but also contributes to students' development of ethical values and sense of responsibility.

### 2.2. STEAM education and the effect of cultural factors (the case of poland-portugal-turkey)

STEAM (Science, Technology, Engineering, Arts and Mathematics) education offers an interdisciplinary approach to produce solutions to the complex and multidimensional problems of the 21st century. This education model is based on four fundamental principles: creativity, inclusiveness, citizenship and emerging technologies ( Quigley and Herro, 2016). STEAM education aims to enable individuals to develop 21st century skills such as critical and creative thinking, responsible innovation, and computational thinking, while at the same time supporting individual, societal, and planetary well-being ( English and Gainsburg, 2016). However, effective implementation of STEAM education requires consideration of cultural factors, as culture significantly shapes individuals' learning processes and environmental awareness ( Komatsu et al., 2019).

Cultural factors play a critical role in the design and implementation of STEAM education. As education systems are shaped by the cultural and socio-economic contexts of countries, the implementation of STEAM education also differs according to these contexts. In this study, secondary school curricula in countries such as Poland, Portugal and Turkey are analysed and the integration of STEAM education and the influence of cultural factors are clearly visible.

In Poland, it is observed that the curriculum has a science and technology-oriented structure, but arts and creativity are less emphasised within the STEAM framework. This shows that a more traditional STEM approach is dominant in Poland's education system ( Videla-Reyes and Aguayo, 2022). In Portugal, STEAM education is associated with subjects such as art and the protection of cultural heritage, enabling students to learn local cultural values. In Turkey, STEAM education has started to be integrated into the curriculum in recent years, but in practice it has been limited to technology and engineering-oriented projects. However, Turkey's cultural diversity has the potential to encourage the use of local and traditional knowledge systems in STEAM projects ( Yakman and Lee, 2012).

This study revealed how different cultural and educational systems influence students' environmental awareness and ethical understanding. In addition, in Japan, environmental education is an integral part of the curriculum, whereas in some developing countries it is not given enough importance ( Pratson et al., 2021). Similarly, the integration of environmental ethics education into STEAM projects in Poland, Portugal and Turkey varies according to the cultural contexts of the countries. In Poland, environmental ethics is more associated with scientific research and environmental protection projects, while in Portugal it is used as a tool for local communities to contribute to sustainable development goals. In Turkey, environmental ethics education is generally considered within the scope of STEM projects, but it is emphasised that it should be addressed in a broader ethical and cultural context within the STEAM framework ( Komatsu et al., 2019).

These differences are also seen in the distinction between individualistic and collectivist cultures. While individualistic cultures prioritise individual responsibility for environmental problems, collective action and social norms may be more determinant in collectivist cultures ( Milfont and Page, 2013). Therefore, STEAM education should be designed and implemented in accordance with cultural contexts. For example, while collaborative learning methods may be more effective in collectivist cultures, projects that encourage individual creativity may come to the fore in individualist cultures ( Moorman and Blakely, 1995).

By taking cultural differences into account, STEAM education can increase students' awareness of environmental problems and enable them to generate innovative solutions to these problems. For example, Eames and Aguayo emphasise the importance of designing STEAM projects in accordance with cultural contexts to develop ecological literacy ( Aguayo et al., 2023). Similarly, Eglash et al. succeeded in increasing both scientific and cultural awareness of students by integrating the cultural values of local communities into STEM/STEAM projects through ethno-computing applications. Such projects not only improve students' technical skills but also strengthen their environmental ethics in a cultural context ( Eglash et al., 2006).

Consequently, STEAM education can become more effective and inclusive by taking cultural factors into account. The implementation of STEAM education in countries such as Poland, Portugal and Turkey differs due to the influence of cultural contexts and educational systems. STEAM projects designed in accordance with cultural contexts increase both scientific and cultural awareness of students, enabling them to acquire the skills necessary for a sustainable future. In this context, STEAM education not only provides technical knowledge and skills, but also strengthens students' cultural values and understanding of environmental ethics.

## 3. Methodology

### 3.1. Research design

This study was conducted to evaluate the environmental ethics awareness of students at the compulsory education stage. This study was designed using a mixed method approach. The study includes both quantitative and qualitative data collection and analysis methods.

The research is conducted in two stages: (1) Document Analysis, which includes a comparative study of STEAM curricula in Poland, Turkey and Portugal, an analysis of their educational philosophies, and an evaluation of environmental ethics education approaches; and (2) Experimental Application, where a STEAM-based environmental ethics education program is tested with students aged 10-14, followed by an assessment of its effectiveness.:

The Environmental Ethics Awareness Scale ( Karakaya and Yılmaz, 2017) instrument was employed. This consists of 23 items answered in a 5-point Likert scale from *Strongly disagree* (1) to *Strongly agree* (5). The scale is structured into four factors: Definition of Environmental Ethics, Environmental Ethics Measures, Reasons for the Emergence of Environmental Ethics and Purpose of Environmental Ethics. The internal consistency of the total score was very good, α = 0.95, ranging from 0.67 (Purpose of environmental ethics) to 0.92 (Definition of environmental ethics).

This research examines the awareness of environmental ethics and the achievements of STEAM (Science, Technology, Engineering, Arts, and Mathematics) education for students aged 10-14 in Portugal, Turkey, and Poland. A content-based analysis method was utilized to compare the curriculum and policy documents of each country ( Table 1).

**Table 1.** Key sources and official frameworks.

Country	Frameworks
**Portugal**	○ Basic Law of the Education System no. 46/86 and no. 16/2023 ○ Decree-Law no. 55/2018 ○ Student Profile at the End of Compulsory Schooling ○ National Strategy for Citizenship Education ○ National Strategy for Environmental Education ○ Action Plan for the Digital Transition of Portugal
**Turkey**	○ Ministry of Education - Middle School Programs ○ Official Documents on STEAM Education in Turkey ○ Science Course Curriculum (Elementary and Middle School Grades)
**Poland**	○ National Curriculum Documents for Compulsory Education ○ Curriculum Regulations of the Polish Ministry of Education ○ Reform Reports on Environmental Education and STEAM Integration ○ Official Documents on Projects and Grant Programs

The analysis began with examining how STEAM education and environmental ethics are integrated into each country's curriculum, focusing on curriculum structure, subject scope, interdisciplinary projects, and the relationship between arts and environmental awareness. Relevant laws, regulations, and strategic plans (e.g., Decree-Law no. 55/2018 in Portugal, MEB Middle School Programs in Turkey, national curriculum reforms in Poland) were analyzed to compare the countries' priorities and implementation methods.

### 3.2. Participants and data collection methods

A total of 283 students in compulsory education from three different countries (Portugal, Turkey and Poland) participated in the study. All participating students were drawn from schools belonging to partner institutions of the Erasmus+ project “Eco-Maker” (KA220-SCH), which focuses on STEAM education and climate change. According to the gender factor, the groups were divided into male and female, and according to the country factor, three groups were formed as Portugal, Turkey and Poland. Data were collected through a scale measuring environmental ethics awareness and students' views on four main factors related to environmental ethics were evaluated. The questionnaire was translated into Portuguese and Polish using a forward–backward translation process. Two bilingual translators independently produced forward translations, which were reconciled by a panel of three subject-matter experts in education. Although rigorous translation procedures were implemented, measurement invariance across countries was not formally tested.

Data were coded using content analysis, identifying common themes and differences in STEAM and environmental ethics practices. The cultural and educational contexts were also analyzed descriptively to understand the delivery of environmental ethics education. This ensured a comprehensive comparison of STEAM-based environmental ethics practices in Portugal, Turkey, and Poland.

During the research process, ethical rules were followed, approval of the research ethics committee was obtained, parental and student permissions were obtained, confidentiality and data security of the participants were ensured. In addition, care was taken to protect the rights of the participants.

Independent sample t-test and MANOVA (Multivariate Analysis of Variance) methods were used to analyse the data. Independent sample t-test was applied to evaluate the general awareness levels of students about environmental ethics. MANOVA was used to examine the effects of gender and country factors on environmental ethics awareness. In addition, significant differences between countries were determined by performing post hoc analyses.

In this study, limitations such as geographical constraints, time limitations, language differences and cultural adaptation requirements were taken into consideration.

This methodology aims to measure both the cross-cultural comparative dimension of the research and the impact of STEAM education on environmental ethical awareness. The mixed method approach will provide more comprehensive answers to the research questions.

## 4. STEAM education and environmental ethics in different countries

### 4.1. The role of education systems

Education systems play a critical role in shaping students' environmental ethics and sustainability awareness. Williams and Chen's research reveals how differences in education systems affect students' understanding of environmental ethics ( McLure et al., 2022). In this context, when the education systems of 10-14 year old in countries such as Poland, Portugal and Turkey are analysed, it is seen that the integration and implementation of STEAM-based environmental education varies according to the cultural and socio-economic contexts of the countries.

In Poland, a science and technology-orientated approach is adopted in the secondary school curriculum, and environmental ethics education is usually covered within science courses. This shows that a more traditional STEM approach is dominant in Poland's education system ( Videla-Reyes, and Aguayo, 2022). For example, in Poland, students are encouraged to develop solutions to environmental problems by using scientific research methods in environmental protection projects. However, the fact that elements such as art and creativity are less involved in these projects limits the full implementation of STEAM education.

In Portugal, STEAM education allows students to learn about local cultural values, especially in relation to subjects such as art and the protection of cultural heritage. This approach encourages students to develop more sensitive and creative solutions to environmental problems. For example, in Portugal, students develop projects for the protection of local ecosystems, while at the same time learning to contribute to the preservation of cultural heritage and sustainable development goals ( Martins, 2017). This shows that STEAM education is not only focused on science and technology, but also includes cultural and artistic dimensions.

In Turkey, STEAM education has started to be integrated into the curriculum in recent years, but in practice it has been limited to technology and engineering orientated projects. However, Turkey's cultural diversity has the potential to encourage the use of local and traditional knowledge systems in STEAM projects ( Yakman and Lee, 2012). For example, students in Turkey increase their environmental awareness through projects such as designing energy efficient house models from recycled materials. However, more inclusion of art and cultural elements in these projects can provide a more inclusive implementation of STEAM education.

Miller's study shows that students who receive STEAM-based environmental education are more sensitive to environmental problems, are more creative in producing sustainable solutions and have a higher awareness of social responsibility ( Larson and Miller, 2011). In this context, the fact that students in Portugal develop creative solutions to environmental problems by learning local cultural values, students in Poland participate in environmental protection projects using scientific research methods, and students in Turkey increase their environmental awareness through recycling projects reveal the different application forms of STEAM education across countries ( Quigley and Herro, 2016).

The education systems of these three countries show different approaches to how environmental ethics education is addressed within the STEAM framework. In Poland, environmental ethics education is mostly associated with scientific research and environmental protection projects, while in Portugal it is used as a tool for local communities to contribute to sustainable development goals. In Turkey, environmental ethics education is generally addressed within the scope of STEM projects; however, it is emphasised that the sustainability dimension of science education in the Anthropocene era should be evaluated in a broader ethical and cultural context within the framework of STEAM approach ( Gülhan, F. 2023).

In conclusion, education systems in countries such as Poland, Portugal and Turkey show differences in the implementation of STEAM education. In Poland, a more traditional STEM approach is adopted, while in Portugal, elements such as art and the preservation of cultural heritage come to the fore. In Turkey, STEAM education is limited to technology and engineering orientated projects but has the potential to become more inclusive by integrating cultural diversity into these projects. These differences play an important role in shaping students' understanding of environmental ethics and sustainability awareness. Designing education systems in a more inclusive and culturally relevant way in this context will allow students to develop more sensitive and creative approaches to environmental problems.

### 4.2. Educational philosophies and student participation

Educational philosophies in different countries have a significant impact on the implementation of STEAM education. In Western countries more student-centred and discovery-based learning approaches are adopted, while in Eastern countries more traditional and teacher-centred approaches are preferred ( Sterling, 2001). These differences influence how students develop environmental ethical awareness through STEAM education. Student-centred approaches develop critical thinking and problem-solving skills while encouraging students' active participation ( Bevan, 2017). This helps students to find more creative and effective solutions to environmental problems.

STEAM education not only raises individual awareness but also acts as a catalyst for societal change. When students learn about environmental issues, they can have a positive impact on their families and friends. This contributes to the spread of environmental awareness throughout society ( Chawla, 1999). For example, environmental projects organised by students in their schools play an important role in raising community awareness and sensitivity to environmental issues ( Lumis, 1998).

### 4.3. STEAM education and environmental ethics outcomes in polish curricula for 10-14 year old

The Polish education system has been modernised with the reforms carried out after the accession to the European Union and has taken important steps especially in the integration of environmental education and STEAM (Science, Technology, Engineering, Arts and Mathematics) education. The curriculum for students aged 10-14 aims to increase students' environmental awareness and develop sustainability awareness by combining scientific and ethical values. The general secondary education curriculum in Poland provides a structure for developing students' scientific, creative and ethical skills through both basic and advanced courses. The curriculum can be adapted to the needs of students and local conditions, giving flexibility to teachers. In this context, the outcomes of STEAM education and environmental ethics can be summarised as follows:

• STEAM Education and Environmental Ethics Integration

In Poland, STEAM education is associated with environmental ethics and students are encouraged to develop scientific and creative solutions to environmental problems. Environmental problems are addressed in science, technology and art courses, and active participation of students is ensured through project-based learning. For example:

• **Science Lessons**: Issues such as environmental pollution, climate change and the protection of natural resources are shared with students and they are expected to develop scientific solutions to these problems. Physics, chemistry and biology courses enable students to analyse environmental problems with scientific methods ( Kolanowska, 2024)
• **Art and Design**: As part of STEAM education, creative projects are designed from recycled materials in art classes. This improves students' environmental awareness and creativity ( Aguayo et al., 2023).
• Environmental Ethics Training

Environmental ethics education in Poland aims to develop students' sense of responsibility towards nature and to approach environmental problems from an ethical point of view. These outcomes are:

• **Respect for Nature and Responsibility**: Students acquire ethical values in nature conservation and sustainability. This is in line with the goal of raising democratic, tolerant and solidaristic individuals ( Dzwonkowska, 2017).
• **Local and Global Environmental Issues**: The curriculum encourages students to understand and develop solutions to both local environmental problems and global environmental crises. Geography and biology subjects support these outcomes ( Kolanowska, 2024).
• Project Based Learning

The Polish education system adopts the project-based learning method, enabling students to actively participate in STEAM education and environmental ethics. Examples

• **Environmental Projects**: Projects are developed on topics such as protection of water resources, energy saving and recycling, which enable students to use scientific and ethical values together ( Dzwonkowska, 2017).
• **Technology and Engineering**: Students are encouraged to develop environmentally friendly technologies and innovative engineering solutions. The integration of maths, physics and chemistry courses supports these outcomes ( Wiśniewski and Zahorska, 2020).

In Poland, teachers are given flexibility in the implementation of the curriculum. In accordance with the general framework set by the Ministry of Education, teachers can adapt course content and materials to the needs of students. For example, school principals and teachers can select advanced courses and organise additional courses according to students' interests ( Kolanowska, 2024).

**Evaluation and Measurement**

In Poland, placement exams after the second stage of primary school are used to measure students' environmental knowledge and STEAM skills. These exams aim to determine students' level of knowledge and evaluate the effectiveness of the curriculum. However, these exams do not aim at placement in higher education as in Turkey ( Şükrü et al., 2016).

After Poland's accession to the European Union, important reforms have been carried out in the education system in terms of environmental education and STEAM integration. The grant programmes and projects provided by the EU aimed to reach schools, especially in rural areas, and to ensure equal opportunities in education. These reforms made it possible for environmental ethics and STEAM education to reach a wider range of students ( Wiśniewski and Zahorska, 2020).

Polish curricula for 10-14-year-olds aim to develop students' scientific, creative and ethical skills in STEAM education and environmental ethics ( Table 2). Flexible and project-based approaches increase students' awareness of environmental problems and enable them to create innovative solutions to these problems. The Polish education system, which has been modernised with the influence of the European Union, offers an exemplary structure for other countries in the integration of environmental ethics and STEAM education ( Kolanowska, 2024).

**Table 2.** STEAM education and environmental ethics outcomes in the Polish curriculum for students aged 10–14.

Acquisition	Courses	Time information	Source
To be able to analyse environmental problems using scientific methods	Physics, Chemistry, Biology	Physics (4 hours/week),Chemistry (4 hours/week), Biology (4 hours/week)	( European Commission, 2024; Kolanowska, 2024)
To be able to develop creative solutions using technological tools	Use of technology integrated with Information Technologies, Modern Foreign Languages	Information Technologies (3 hours/week)	( Rozporządzenie Ministra Edukacji, 2024)
To be able to design sustainable projects by applying engineering principles	Integration of Maths, Physics and Chemistry	Maths (14 hours/4 annual cycle)	( Rozporządzenie Ministra Edukacji, 2024)
To be able to produce projects that increase environmental awareness through art	Visual Arts, Music	Visual Arts (1 hour/week, elective), Music (1 hour/week, elective)	( Rozporządzenie Ministra Edukacji, 2024)
Analysing environmental data with mathematical skills	Mathematics, Geography	Maths (3-4 hours/week), Geography (4 hours/4-year cycle)	( Dzıennık Ustaw Rzeczypospolıtej Polskıej, 2017)

**Environmental Ethics Gains**

The curriculum integrates environmental ethics across multiple subjects to foster responsibility, sustainability, and ethical decision-making ( Table 3). Biology and Geography teach responsibility towards nature (4 hours per 4-year cycle), while Business and Management, Geography, and Biology cover sustainability principles (2 hours in Business and Management). Geography and History address local and global environmental issues (1-2 hours per week in Geography, 7 hours per 4-year cycle in History). Ethical environmental decision-making is explored in Philosophy, Religion, or Ethics (1 hour per week in Philosophy as an elective). Additionally, eco-friendly practices are promoted through teacher-guided activities and school-approved lessons (4 hours per 4-year cycle). This framework follows the Rozporządzenie Ministra Edukacji (2024).

**Table 3.** Poland environmental ethics gains.

Acquisition	Courses	Time information	Source
Developing a sense of responsibility towards nature	Biology, Geography	Biology (4 hours/4-year cycle), Geography (4 hours/4-year cycle)	( Rozporządzenie Ministra Edukacji, 2024)
Understanding and applying the principles of sustainability	Integration of Business and Management, Geography and Biology	Business and Management (2 hours)	( Rozporządzenie Ministra Edukacji, 2024)
Evaluation of local and global environmental problems	Geography, History	Geography (1-2 hours/week), History (7 hours/4-year cycle)	( Rozporządzenie Ministra Edukacji, 2024)
To be able to make environmental decisions within the framework of ethical values	Philosophy, Religion or Ethics (elective course)	Philosophy (1 hour/week, elective)	( Rozporządzenie Ministra Edukacji, 2024)
Encouraging environmentally friendly practices	Class teacher-guided activities, additional lessons approved by the school council	Classroom activities (4 hours/4 annual cycle)	( Rozporządzenie Ministra Edukacji, 2024)

### 4.4. STEAM education and environmental ethics gains for 10-14-year-olds in the portuqal education system

In line with the sustainable development goals and the environmental policies of the European Union, the Portuguese education system has been integrating STEAM (Science, Technology, Engineering, Arts, and Mathematics) education and environmental ethics into its curriculum. In the 2nd and 3rd cycles of basic education (covering roughly the 10-14 age group), environmental education and STEAM outcomes are introduced through various subjects and official guidelines. These outcomes aim to enhance students’ environmental awareness, foster sustainability consciousness, and encourage scientific, technological, and creative thinking skills.

4.4.1 STEAM Education Gains

1. **Scientific and Technological Awareness**: In Natural Sciences and Mathematics, students examine issues like environmental pollution and climate change, analyze real-life data, and propose scientific solutions. This approach develops an understanding of how scientific methods and technological innovations can address global and local ecological challenges ( Government of Portugal, 2018a, 2018b).
2. **Environmental Awareness through Art and Creativity:** Art and interdisciplinary projects often involve the use of recyclable materials to create artistic works, thereby increasing both creativity and environmental sensitivity ( Martins, 2017). By connecting artistic expression with ecological concerns, students learn new ways to reflect on sustainability ( Portuguese Environment Agency (APA), 2020).
3. **Analysing Environmental Data with Mathematical Skills:** Through Mathematics lessons, learners develop the skills to collect, interpret, and evaluate environmental data. For example, students may analyze air or water quality statistics to identify trends and propose solutions based on quantitative evidence ( Portuguese Environment Agency (APA), 2020). This fosters a deeper understanding of environmental issues and the role of mathematical modeling in informing policy and personal decisions.
4. **Assessing Scientific and Technological Impacts on Sustainability:** In Citizenship and Development or Social and Civic Education courses, students discuss how scientific discoveries and technological applications affect the environment ( Government of Portugal, 2025). They also explore specific threats, such as habitat destruction or pollution, and debate the necessity of responsible research and innovation.

4.2.2. Environmental Ethics Gains ( Government of Portugal, 1986, 2018a, 2018b)

1. **Respect for Nature and Awareness of Responsibility:** Environmental ethics education teaches students to approach ecological problems with a heightened sense of personal and collective responsibility. In Natural Sciences, topics like deforestation and biodiversity loss are used to illustrate ethical imperatives around stewardship of the environment ( Portuguese Environment Agency (APA), 2020).
2. **Local and Global Environmental Problems:** Learners are exposed to environmental issues at multiple scales, from waste disposal in their own communities to global challenges like climate change ( Portuguese Environment Agency (APA), 2020). By exploring these contexts, they develop empathy and problem-solving skills that account for diverse impacts and solutions.
3. **Environmental Decision Making within the Framework of Ethical Values:** Through project-based learning and group discussions, students cultivate the ability to make decisions guided by ethical principles ( Government of Portugal, 2025). They examine dilemmas relating to overconsumption, resource allocation, and responsible innovation, grounding their conclusions in respect for human well-being and ecological balance.
4. **Prevention of Environmental Risks:** Practical activities—such as laboratory experiments or field studies—help learners assess and mitigate environmental hazards, leading them to understand the need for preventive measures ( Portuguese Environment Agency (APA), 2020). By evaluating real or simulated scenarios (e.g., water contamination), students acquire proactive strategies for preserving ecosystems ( Government of Portugal, 1986).

4.2.3. General Framework of the Curriculum

Portugal’s core educational documents ( Government of Portugal, 2018a, 2018b, 2020), emphasize the integration of environmental ethics and STEAM education across curricular areas. The Student Profile at the End of Compulsory Schooling guide ( Government of Portugal, 2017) underscores the development of critical thinking, problem-solving, and innovation mindsets. These guidelines encourage:

• Interdisciplinary Collaboration: Linking concepts in science, technology, arts, and mathematics to real-world ecological challenge ( Government of Portugal, 2018a)
• Practical and Contextual Learning: Employing active methodologies (e.g., experiments, projects involving local community) to foster ecological literacy and civic engagement ( Government of Portugal, 2017).
• Ongoing Assessment: Continuously revisiting and assessing students’ ecological knowledge, awareness, and skills as they progress through the 2nd and 3rd cycles of basic education ( Government of Portugal, 1986).

Through these provisions, the Portuguese education system ( Table 4) seeks to cultivate learners who are scientifically literate, ethically grounded, and motivated to engage with and address the ecological challenges of the modern World ( Portuguese Environment Agency (APA), 2020).

**Table 4.** Compact summary of STEAM education, environmental ethics, and curriculum framework acquisitions in Portuguese curriculum policy documents for compulsory schooling (ages 10–14). The full version of Table 4 (complete extraction/coding of curriculum statements) is available as supplementary material in Zenodo ( Arce, and Güler, 2026; DOI: 10.5281/zenodo.18590541).

Field	Key acquisitions (ages 10–14)	Key acquisitions (ages 10–14)
STEAM – Scientific/technological and engineering awareness	Analyse environmental issues (pollution, climate change, resource conservation) using scientific methods; apply engineering principles towards environmentally friendly solutions/technologies.	Basic Law on the Education System (Law No. 46/86); Decree-Law No. 55/2018
STEAM – Arts and creativity	Design creative projects (e.g., with reusable/recyclable materials) to foster environmental awareness and artistic sensitivity.	Basic Law on the Education System (Law No. 46/86)
Environmental ethics – Responsibility and respect for nature	Develop responsibility toward nature and frame environmental challenges through an ethical/citizenship lens.	National Strategy for Citizenship Education; National Strategy for Environmental Education (APA); Law No. 46/86
Curriculum framework – Digitalisation and sustainable development	Build digital skills to collect/share/critically evaluate environmental data (e.g., sensors/apps); integrate sustainable development goals to promote local–global environmental responsibility.	Action Plan for Portugal’s Digital Transition; Decree-Law No. 55/2018; Citizenship Education Strategy; Environmental Education Strategy (APA)

### 4.5. STEAM education and environmental ethics gains for 10-14-year-olds in turkish education system

The integration of STEAM education and environmental ethics in Turkey is undergoing a significant transformation within the scope of the 2023 Education Vision ( Government of Turkey, 2019). The curriculum, especially at the secondary school level (10-14 years old), aims to integrate the fields of science, technology, engineering, arts, and mathematics with environmental sustainability goals through an interdisciplinary approach ( Government of Turkey, 1973 and 2019). Unlike the traditional STEM model, the STEAM approach in Turkey offers a holistic perspective including artistic creativity and cultural values and supports environmental ethics ( Akpinar and Akgunduz, 2022)

In the Turkish education system, environmental ethics education ( Table 5) is generally addressed within the scope of STEM projects; however, it is emphasized that the sustainability dimension of science education in the Anthropocene era should be evaluated in a broader ethical and cultural context within the framework of STEAM approach ( Akaydın and Eşme, 2024). In this context, in line with Turkey's 2053 Net Zero Emission targets, integrating environmental awareness and sustainability education with the STEAM approach is considered as a strategic priority ( Government of Turkey, 2023).

**Table 5.** Compact summary of STEAM achievements and environmental ethics gains across course areas in the turkish middle school curriculum (ages 10–14). The full version of Table 5 is available as supplementary material in Zenodo ( Arce and Güler, 2026; DOI: 10.5281/zenodo.18590541).

Course area	STEAM achievements	Environmental ethics gains	Main source
**Science**	Scientific method; problem solving; energy efficiency & renewables; science-based analysis of pollution/climate/resource conservation.	Awareness of global environmental problems; sustainability and pro-environmental behaviour.	Turkish Ministry of National Education. ( 2018a)
**Mathematics**	Modelling & data analysis; interpretation of environmental indicators (e.g., emissions/energy use).	Quantitative reasoning for environmental problem-solving and sustainable resource management.	Turkish Ministry of National Education. ( 2018a, 2018b)
**Technology & Design**	Engineering design process; eco-friendly tools/projects; recycling and green technologies in everyday life.	Environment-sensitive design; evaluation of environmental impacts of technology.	Turkish Ministry of National Education. ( 2018a)
**Visual Arts**	Creative projects with recycled materials; environmental-themed artistic production.	Environmental awareness through art; respect for nature and eco-visual communication.	Gov. of Turkey (2012)
**Information Technologies & Software**	Algorithmic thinking & coding; digital production and use of ICT for environmental data/tasks.	Responsible digital practices supporting sustainability and environmental awareness.	Turkish Ministry of National Education. ( 2018b)

## 5. Data analysis

In this study, the environmental ethics awareness levels of students at the compulsory education stage were analysed and it was found that students exhibited a high level of awareness of four main factors related to environmental ethics (Definition of Environmental Ethics, Environmental Ethics Measures, Reasons for the Emergence of Environmental Ethics and Purpose of Environmental Ethics). A total of 283 students from Portugal, Turkey and Poland participated in the study. Of the total sample, 163 students were from Portugal (57.6%), 86 from Poland (30.4%), and 34 from Turkey (12.0%). In terms of gender distribution, the sample was composed of 52.7% woman (n = 149) and 47.3% men (n = 134). A scale developed to measure environmental ethics awareness was applied to the participants. This scale assessed four main factors related to environmental ethics: Definition of Environmental Ethics, Environmental Ethics Measures, Reasons for the Emergence of Environmental Ethics and Purpose of Environmental Ethics.

Mean comparison tests were executed. One-sample t test to compare the mean of the distribution with a test value (neither agree or disagree) was used, and the magnitude of the effect was measured in Cohen’s d (Hedge’s formula) being classified as small ( *d* = 0.20), medium ( *d* = 0.50), large ( *d* = 0.80) and more than large ( *d* = 1.20) ( Vilariño et al., 2022; Cohen, 1988). As for the mean comparisons of multiple dependent related variables (dependent variables encompass a latent variable, environmental ethics) for a two (gender: female vs. male) and three level factor (country: Portugal, Turkey, Poland), MANOVAs were performed. Assumption of homogeneity was violated for the country factor (Box’ M was significant). This contingency may cause important deviations in the significance (p) associated to *F* values (empirical *F*s). Then, Pillai’s trace was computed for multivariate *F*s due to its robustness to the heterogeneity effects of the variance matrices. As for validating the acceptance of the alternative hypothesis in univariate *F*s three safeguards were verified ( Mayorga et al., 2020): the empirical *F* was greater than the theoretical (df *(*1, N-k/k), where k is the number of levels of the factor; the observed effect size was ≥ small magnitude; and that the probability of false acceptance of the null hypothesis was equal o higher than the probability of false acceptance of the alternative hypothesis (β/α ≥ 1). Effect size for numerator *df* > 1 were computed as η ^2^ (small: η ^2^ = .001; medium: η ^2^ = .059; large: η ^2^ = .138; and η ^2^ = .138; Arce et al., 2015: Cohen et al., 1988) and as Cohen’s *d* for numerator *df* = 1 (small: *d* = 0.20; medium: *d* = 0.50; large: *d* = 0.80; and more than large: *d* = 1.20; Arce et al., 2015). Post hoc analysis were analyzed with Sidak’s correction (equal variance and unequal sample size; *N*_large size_/ *N*_low size_ > 1.5) or Dunnett’s C (unequal variance and unequal sample size). Effect size magnitudes were quantified calculating the probability of Superiority of the Effect Size (PS _ES_; Vilariño et al., 2022). This lets the interpretation of the effect as a percentile.

## 6. Results

The results of the study of environmental ethics awareness in the population of students in compulsory education stage showed that they agree with the definition of environmental ethics, *t*(282) = 39.80, *p* < .001, with more than large effect size, *d* = 2.39, above 95.45% of the possible effects, PS _ES_ = .9545; with the environmental ethics measures to be taken, *t*(282) = 35.17, *p* < .001, with more than large effect size, *d* = 2.08, above 92.92% of the possible effects, PS _ES_ = .9292; with the emergence of environmental ethics reasons, *t*(282) = 28.76, *p* < .001, with more than large effect size, *d* = 1.59, above 86.86% of the possible effects, PS _ES_ = .8686; and with purpose of environmental ethics *t*(282) = 30.96, *p* < .001, with more than large effect size, *d* = 1.71, above 88.69% of the possible effects, PS _ES_ = .8869.

### 6.1. Comparison of environmental ethics awareness levels between countries

The results of a MANOVA on the environmental ethics awareness exhibited significant differences, Pillai’s Trace = .113, *F*(8, 556) = 4.15, *p* < .001, 1–β = .994, explaining 5.6% of the variance, η ^2^ = .056, a medium magnitude effect size higher than 63.68% of the effects, PS _ES_ = .6368, for the country factor (Portugal, Turkey, Poland).

The univariate effects revealed a significant effect on the Definition of Environmental Ethics for the country factor, *F*(2, 280) = 10.99, *p* < .001, 1–β = .991, explaining 7.3% of the variance, η ^2^ = .073, a medium magnitude effect higher than 65.54 of the effects, PS _ES_ = .6554. Post hoc analysis displayed that Portugal ( *M* = 31.75) students agreed significantly more with the definition of Environmental Ethics than Turkey ( *M* = 28.71) and Poland ( *M* = 29.97) students, with a large, *d* = 0.82 (above 71.90% of all possible, PS _ES_ = .7190), and a close to medium, *d* = 0.45 (above 62.55% of all possible, PS _ES_ = .6255), effect size magnitude, respectively.

The univariate effects also exhibited a significant effect on the Emergence of Environmental Ethics Reasons for the country factor, *F*(2, 280) = 6.58, *p* = .002, 1–β = .908, explaining 4.5% of the variance, η ^2^ = .045, an small effect higher than 61.79% of all effects, PS _ES_ = .6179. Post hoc analysis displayed that Portuguese ( *M* = 21.67) students agreed significantly more with the Emergence of Environmental Ethics Reasons Factor than Turkish ( *M* = 19.88) and Polish ( *M* = 20.30) students, with a medium, *d* = 0.57 (above 65.54% of all possible effects, PS _ES_ = .6554), and a low, *d* = 0.39 (above 61.03% of all possible effects, PS _ES_ = .6103), effect size magnitude, respectively.

The univariate effects exposed a significant effect on the Purpose of Environmental Ethics for the country factor, *F*(2, 280) = 5.85, *p* = .003, 1–β = .871, explaining 4.0% of the variance, η ^2^ = .040, a small magnitude effect higher than 61.41% of the effects, PS _ES_ = .6141. Post hoc analysis displayed that Portuguese ( *M* = 13.38) students agreed significantly more with the Purpose of Environmental Ethics Factor than Polish ( *M* = 12.43) students with a medium, *d* = 0.52 (above 61.41% of all possible, PS _ES_ = .6141), effect size magnitude.

No univariate effect were observed on the Environmental Ethics Measures for the country factor, *F*(2, 280) = 2.50, *p* = .084, η ^2^ = .018, 1–β = .500.

### 6.2. Results according to gender factor

The results of a MANOVA on the environmental ethics awareness showed no differences for the gender factor (male vs. female), Pillai’s Trace = .098, *F*(4, 278) = 0.57, *p* = .686, η ^2^= .008, 1–β = .188. This finding shows that environmental ethics awareness transcends gender boundaries and environmental responsibility awareness is adopted as a common value. This is in line with broader educational goals that emphasise that environmental education should be implemented in an inclusive and equitable manner. The absence of a gender difference indicates that environmental ethics awareness has reached a common level of consciousness among individuals and gender equality has been achieved in this regard.

## 7. Discussion and conclusions

The results of the study reveal that there is a generally high awareness of environmental ethics among students, but this awareness differs between countries. These differences can be considered as a reflection of the educational systems, cultural values, and the importance they attach to environmental ethics. In addition, the fact that there is no significant difference according to the gender factor shows that environmental ethics awareness is a common value that transcends gender boundaries. These findings emphasise that countries should develop strategies appropriate to their own educational and cultural contexts to increase environmental ethics awareness.

The lack of significant differences based on gender reinforces the idea that environmental ethics awareness transcends gender boundaries, promoting a shared sense of responsibility. This finding aligns with broader educational goals of fostering inclusivity and equity in environmental education. However, the significant differences observed between countries highlight the importance of considering cultural, societal, and educational frameworks when addressing environmental ethics on a global scale.

The notably higher levels of agreement among Portuguese students, particularly regarding the Definition of Environmental Ethics, Emergence of Environmental Ethics Reasons, and Purpose of Environmental Ethics, suggest that educational strategies in Portugal may place greater emphasis on these aspects. These results could reflect curricular differences, pedagogical approaches, or broader cultural values emphasizing environmental stewardship. In contrast, the relatively lower agreement observed among students in Turkey and Poland may indicate opportunities to strengthen environmental ethics education in these countries.

Thus, the findings suggest a high level of environmental ethics awareness among students, with country-specific variations that may reflect cultural or educational differences in environmental ethics emphasis. These results highlight the need for tailored approaches to fostering environmental ethics awareness across different educational contexts.

In short, students have developed an extraordinary positive environmental ethics awareness (the effects are over 85% in the underlying dimensions to environmental ethics awareness). The high level of Portuguese students' results in environmental ethics awareness may be an indicator of the importance given to environmental ethics education in this country. The higher awareness of Portuguese students, especially in the factors of Definition of Environmental Ethics, Reasons for the Emergence of Environmental Ethics, and Purpose of Environmental Ethics, suggests that the curriculum in this country emphasises environmental ethics issues more or that pedagogical approaches address these issues more effectively. Additionally, it can also be considered that cultural values in Portugal may have a structure that encourages environmental responsibility and ethical consciousness. As a consequence, Portuguese students have developed a high sensitivity to environmental ethics awareness.

In contrast, the relatively lower environmental ethics awareness of students in Turkey and Poland suggests that environmental ethics education in these countries needs to be strengthened. This indicates that environmental ethics topics should be included more in the curriculum, teaching methods should be improved, and cultural and social initiatives to raise environmental awareness should be encouraged.

## Ethics and consent

The study was conducted in accordance with the ethical principles of the World Medical Association Declaration of Helsinki – Ethical Principles for Medical Research Involving Human Participants (last amended by the 75th WMA General Assembly, Helsinki, Finland, October 2024), and with the research ethics and data protection guidelines of Bursa Technical University. The study protocol was reviewed and approved by the Bursa Technical University Science, Engineering and Social Sciences Research Ethics Committee (Fen, Mühendislik ve Sosyal Bilimleri Araştırmaları Etik Kurulu), approval ref. E-69707128-050.04-175216 (Meeting No. 2024-21, Decision No. 2, 08 November 2024). This approval covered the full study protocol implemented across the three participating countries (Portugal, Poland and Turkey). The research consisted of an anonymous opinion questionnaire administered to students aged 10–14 years in school settings and posed no more than minimal risk to participants.

In Portugal and Poland, the participating schools do not have institutional ethics committees and, under national regulations, formal ethics committee approval is not legally required for anonymous, minimal-risk, school-based educational research outside clinical/biomedical contexts. In both countries, the study was formally authorised by the school management. Written informed consent was obtained from parents or legal guardians of all participating students, together with assent from the students themselves, prior to questionnaire administration.

In Turkey, in addition to the ethics committee approval described above, the participating school authorised the study, and written informed parental consent and student assent were obtained prior to data collection. Across all sites, participation was voluntary, students were informed that they could withdraw at any time without any negative consequences, and all questionnaire data were collected, stored, and analysed anonymously in aggregated form (no identifying personal data were collected).

## Data Availability

The dataset underlying this article is available in Zenodo and is distributed under the terms of a Creative Commons Attribution 4.0 International licence. The data file(s) include:
i) the anonymised item-level responses to the Environmental Ethics Awareness Scale, ii) the values used to compute descriptive statistics and inferential analyses reported in the article, and iii) the values used to construct all tables and figures. The dataset is openly accessible at Zenodo: https://doi.org/10.5281/zenodo.17477720 ( Arce, and Güler, 2025). Supplementary material, including the full version of large tables (i.e., Table 4, Table 5), is available in Zenodo ( Arce and Güler, 2026): https://doi.org/10.5281/zenodo.18590541. The dataset underlying this article is available in Zenodo and is distributed under the terms of a Creative Commons Attribution 4.0 International licence.
