# Supplementary material for: STEAM education and environmental ethics awareness among students aged 10-14 from an international perspective: a comparative analysis of cultural and educational philosophy factors
Source: Open Res Eur. 2026 Apr 10;6:103. [Version 1] doi: 10.12688/openreseurope.21790.1 (PMC13150443; doi:10.12688/openreseurope.21790.1)
Supplement: Peer review history

# Reviewer report: Reviewer response for version 1

**DOI:** 10.21956/openreseurope.23570.r72299

**Published:** 2026-05-06

**Authors:** José Manuel dos Santos dos Santos

**Affiliations:** 1 Department of Mathematics, University of Coimbra, Coimbra, Portugal

This article present the research developed from a study that pretends analyse Environmental ethics awareness in 3 countries were students was involved in a project using STEAM and other active learning activities.

 The text are well written, the data analyses methods are consistent and allows the researchers do the presented conclusions.

 However, im my opinion, could be include some more actual references in this field of research namely related with STEAM issues.

Is the study design appropriate and does the work have academic merit?

Yes

Is the work clearly and accurately presented and does it cite the current literature?

Partly

If applicable, is the statistical analysis and its interpretation appropriate?

Yes

Are all the source data underlying the results available to ensure full reproducibility?

Yes

Are the conclusions drawn adequately supported by the results?

Yes

Are sufficient details of methods and analysis provided to allow replication by others?

Yes

Reviewer Expertise:

Mathematics (Computational Algebra); Mathematics Education.

I confirm that I have read this submission and believe that I have an appropriate level of expertise to confirm that it is of an acceptable scientific standard.

# Reviewer report: Reviewer response for version 1

**DOI:** 10.21956/openreseurope.23570.r72297

**Published:** 2026-05-06

**Authors:** Nosheen Malik

**Affiliations:** 1 Department of Education, The Islamia University of Bahawalpur, Bahawalpur city, Punjab, Pakistan

This article describes an important topic by comparing the relationship between STEAM education and environmental ethics awareness especially among students aged 10-14.  The appropriate statistical analysis is used such as MANOVA and t-test. The comparative analysis between Portugal, Turkey and Poland is valuable which highlighting how cultural and curricular differences influence in developing environmental ethics. However study could be further improved by having more detailed sampling. By doing this it could have produced more better and generalized results.

 Overall this article is a wonderful contribution in the field of sustainable education and it can give meaningful insight for educators and Policy makers to inter-relate ethical and environmental paradigms into STEAM education

Is the study design appropriate and does the work have academic merit?

Yes

Is the work clearly and accurately presented and does it cite the current literature?

Yes

If applicable, is the statistical analysis and its interpretation appropriate?

Yes

Are all the source data underlying the results available to ensure full reproducibility?

Yes

Are the conclusions drawn adequately supported by the results?

Yes

Are sufficient details of methods and analysis provided to allow replication by others?

Partly

Reviewer Expertise:

AI in education, career counselling, steam education, educational psychology

I confirm that I have read this submission and believe that I have an appropriate level of expertise to confirm that it is of an acceptable scientific standard.
